# Changes in interleukin-27 levels in patients with acute coronary syndrome and their clinical significance

**DOI:** 10.7717/peerj.5652

**Published:** 2019-01-04

**Authors:** Lin Zhang, Junfeng Zhang, Shaohong Su, Suyan Luo

**Affiliations:** Department of the Fifth Affiliated Hospital, Zhengzhou University, Zhengzhou, China

**Keywords:** IL-27, Th2 cells, Acute coronary syndrome

## Abstract

**Background:**

This study evaluated changes in interleukin (IL)-27 levels in patients with acute coronary syndrome (ACS) and their influence on Th1, Th2, and Th17 cells.

**Methods:**

Serum levels of IL-27, IL-4, IL-17, and interferon (IFN)-γ in healthy subjects as well as patients with ACS, including stable angina pectoris (SA), unstable angina pectoris (UA), and acute myocardial infarction (AMI), were determined using an enzyme-linked immunosorbent assay. The proportions of Th1, Th2, and Th17 cells among peripheral blood mononuclear cells (PBMCs), were measured using flow cytometry, after incubation with phorbol myristate acetate (PMA) for 4 h. The proportions of Th1 and Th17 cells among PBMCs in AMI and UA were detected after stimulation with IL-27 or PMA + IL-27 for 4, 8, and 12 h.

**Results:**

Serum levels of IL-27 in patients with AMI and UA were significantly lower than those in SA and control groups, while serum levels of IL-17 and IFN-γ in AMI and UA groups were dramatically increased compared to those in SA and healthy control groups. However, there were no statistically significant differences in serum IL-4. The proportions of Th1 and Th17 cells among PBMCs were statistically significantly higher in the AMI and UA groups than those in the SA and control groups, while there was no statistically significant difference in the proportion of Th2 cells among different groups. For patients with AMI and UA, the effect of co-stimulation of PBMCs with PMA and IL-27 was not significantly different from that of PMA single stimulation, while PMA + IL-27 co-stimulation lowered the Th17 cell proportion significantly compared to PMA single stimulation.

**Discussion:**

Compared to SA patients and healthy controls, patients with ACS (AMI + UA) had lower serum levels of IL-27 and higher proportions of PBMC Th1 and Th17 cells, which could be attributed to the inhibitory effects of IL-27 on the proliferation of Th17 cells. These results indicated that IL-27 could be a novel therapeutic target in ACS patients.

## Introduction

Coronary heart disease (CHD), also known as coronary atherosclerotic heart disease, is characterized by the stenosis or obstruction of coronary arteries, leading to deficiency of both blood and oxygen in the heart (Sayols-Baixeras et al., 2014). Inflammation of the coronary endothelium is thought to play an important role in the progression of atherosclerosis (AS) (Wang et al., 2017). Acute coronary syndrome (ACS), clinically expressed as unstable angina (UA), acute myocardial infarction (AMI), and sudden death, refers to the occurrence of atherosclerotic plaque rupture, vascular endothelial damage, or plaque fissure development and secondary thrombosis, further leading to complete or incomplete coronary artery occlusion (Fujisue & Tsujita, 2017). Several inflammatory factors promote the development of unstable plaque from stable plaque, which may induce plaque rupture, thrombosis, and complete or incomplete obstruction of the coronary arteries, and finally the occurrence of ACS (Dong et al., 2017; Ma et al., 2017; Wilbert-Lampen et al., 2010).

Interleukin (IL)-27, as a member of the IL-12 family, is the only cytokine present in the form of a heterodimer (Behzadi, Behzadi & Ranjbar, 2016). Previous studies found that IL-27 had a wide range of immunosuppressive effects (Boivin et al., 2015) and played a very important role in a variety of inflammatory diseases (Cui et al., 2017; Nemeth et al., 2017; Olsen et al., 2011). The immunosuppressive attributes of IL-27 on Th17 have been reported to induce STAT3-mediated T cell production of interleukin 10 and direct inhibit GATA3 and RoRγt thereby repressing Th17 (Hunter & Kastelein, 2012). IL-27 was suggested to be related to the development of coronary arterial lesions in patients with Kawasaki disease (Si et al., 2017). However, the role of IL-27 in ACS patients has not yet been reported. Therefore, the present research aimed to identify changes in the level of IL-27 and related immune cells in patients with ACS, as well as the clinical significance of IL-27 in ACS.

## Materials and Methods

### Patients

Patients admitted with chest pain to Zhengzhou University, Fifth Affiliated Hospital between January 2015 and August 2015 were identified, and all participants gave verbal informed consent. History taking, physical examination, serum high-sensitivity troponin testing, electrocardiography (ECG), heart ultrasound, and coronary angiography were performed, and a total of 65 patients met the inclusion criteria. Patients were aged 62  ± 10.6 years, and included 38 males and 27 females who were divided into AMI (19 cases), UA (25 cases), and SA (21 cases) groups. Twenty healthy controls, including 13 males and 7 females with an average age of 59  ± 8.4 years, were enrolled from the hospital physical examination centre. Current study was approved by the hospital Ethics Committee in Zhengzhou University (approval number: 2014-292).

### Inclusion criteria

Diagnosis of AMI according to World Health Organization criteria: typical chest pain lasting longer than 30 min; typical dynamic changes on ECG; and dynamic myocardial enzyme (creatine kinase isoenzyme or troponin) changes, with any two of the above confirmed. UA diagnostic criteria: nearly 48 h of at least one episode of resting or spontaneous angina, without enzyme changes of myocardial necrosis, accompanied by ECG ST depression or T wave changes. SA diagnostic criteria: (1) retrosternal or left side chest pain with radiation to the left neck, shoulder, arm, jaw, or back; (2) occurring during emotional stress or exertion and relieved within several minutes by rest; (3) exaggerated by eating or cold weather.

### Exclusion criteria

Patients with any one of the following criteria were excluded: (1) concurrent liver, kidney, cardiopulmonary, hematopoietic, or other serious primary disease; (2) presence of malignant tumour; (3) complicated by stroke, peripheral vascular disease, or peripheral vascular thrombosis; (4) rheumatoid immune disorder; (5) sexually transmitted disease; (6) taking immunosuppressive drugs; (7) infectious disease such as sepsis; (8) severe upper respiratory tract infection; (9) lung or biliary tract infection; (10) high fever; (11) use of anti-inflammatory medication.

### Blood collection and peripheral blood mononuclear cell (PBMC) isolation

Fasting peripheral venous blood (20 mL) was drawn. PBMC were collected in the cell separation medium (Ficoll; Shanghai Bioscience & Technology, Shanghai, China) and re-suspended (1 × 10^6^ cells) in RPMI 1640 medium (Thermo Fisher Scientific, Shanghai, China), which contained 10% calf serum (Hanyang Biologicals Technology, China). PBMCs were incubated at 37 °C for 24 h for later use.

### TH cell subset analysis

PBMCs were analysed using flow cytometry (FACSVantage SE, BD, NJ, USA). Th1 and Th2 cells subsets were identified based on the detections of CD3 and CD4, as well as intracellular cytokines, IFN-c or IL-4. Briefly, cells were labelled with APC mouse anti-human CD3 and PE-Cy-™5 mouse anti-human CD8, and then stained with FITC-mouse anti-human IFN-c and PE-mouse anti-human IL-4 antibodies (all antibodies were from BD, NJ, USA). Th1 cells were labelled with CD3+ CD8- IFN-c+ and Th2 cells were labelled with CD3+ CD8- IL-4+. Number of cells was reported as a percentage of total CD3+ cells.

### T cell stimulation

T cells were incubated in RPMI 1640 medium containing phorbol myristate acetate (PMA, 25 ng/mL) and ionomycin (1 µg/mL) (Sigma Chemical, USA), as well as NMDA antagonists (ketamine, from Henrui Pharmacology, China and MK-801, from Sigma Chemical Co., USA) in 95%-humidified atmosphere with 5% carbon dioxide at 37 °C. After stimulation for 4 h, supernatants and cells were collected for further studies.

### Enzyme-linked immunosorbent assay

Plasma was centrifuged at 3,000 rpm for 5 min. Plasma IL-27, IL-4, IL-17, and IFN-γ levels were determined using the ELISA method (R & D Systems, Minneapolis, MN, USA), following the instructions from the manufacturers.

### Flow cytometry

A total of 500 µL of whole blood was mixed with an equal volume of RPMI 1640. Mononuclear cells were harvested by density gradient centrifugation in lymphocyte separation media. Cells were divided into a single PMA stimulation group and a PMA + IL-27 stimulation group. In the PMA stimulation group, cells were incubated with ionomycin, PMA, and BD GolgiStop™ Protein Transport Inhibitor for 8 h at 37 °C in 5% CO_2_. In the PMA + IL-27 stimulation group, PMA, ionomycin, BD GolgiStop™ Protein Transport Inhibitor, and IL-27 were added to cell medium and incubated at 37 °C in 5% carbon dioxide for 8 h. Then, 20 µL of the mixed antibody from the Human Th1, Th2, and Th17 phenotyping kit (BD FACSCalibur; BD Biosciences, San Jose, CA, USA) was added to cell group and incubated for 30 min. IFN-γ, IL-4, and IL-17 levels were detected with flow cytometry (BD FACSCalibur), respectively, to reflect the proportion of Th1, Th2, and Th17 cells in patients.

### Statistical analysis

Results were analysed using SPSS 17.0 (SPSS, IBM, USA). Data were expressed as X  ± S, and comparisons among groups were analyzed with single-factor analysis of variance. A *P* < 0.05 was considered statistically significantly different.

## Results

### Baseline patient characteristics

As shown in Table 1, 65 cases with coronary heart disease were analyzed, with 19 cases of AMI, 25 of UA, and 21 of SA. There were 38 males and 27 females, aged 62 ± 10.6 years. There were 20 healthy cases, including 13 males and 7 females, aged 59 ± 8.4 years.

**Table 1:** Baseline characteristics of study participants.

	Control (*N* = 20)	AMI (*N* = 19)	UA (*N* = 25)	SA (*N* = 21)	*P*
Gender					
Male, *N* (%)	13 (65.0%)	11 (57.9%)	15 (60%)	13 (61.9%)	0.127
Age, year, mean ± standard deviation	59 ± 8.4	57 ± 7.5	60 ± 3.8	62 ± 10.6	0.245
Body mass index, kg/m^2^, mean ± standard deviation	20 ± 2.7	20 ± 6.9	22 ± 1.6	21 ± 4.2	0.313
Disease history, *N* (%)					
Hypertension	9 (45%)	9 (47.4%)	11 (44%)	10 (47.6)	0.119
Diabetes	0	0	0	0	
Smoker	7 (35%)	6 (31.6%)	9 (36%)	7 (33.3%)	0.185
Dyslipidemia	6 (30%)	5 (26.3%)	8 (32%)	7 (33.3%)	0.092

### Serum IL-27 levels in different ACS groups

**Figure 1:**
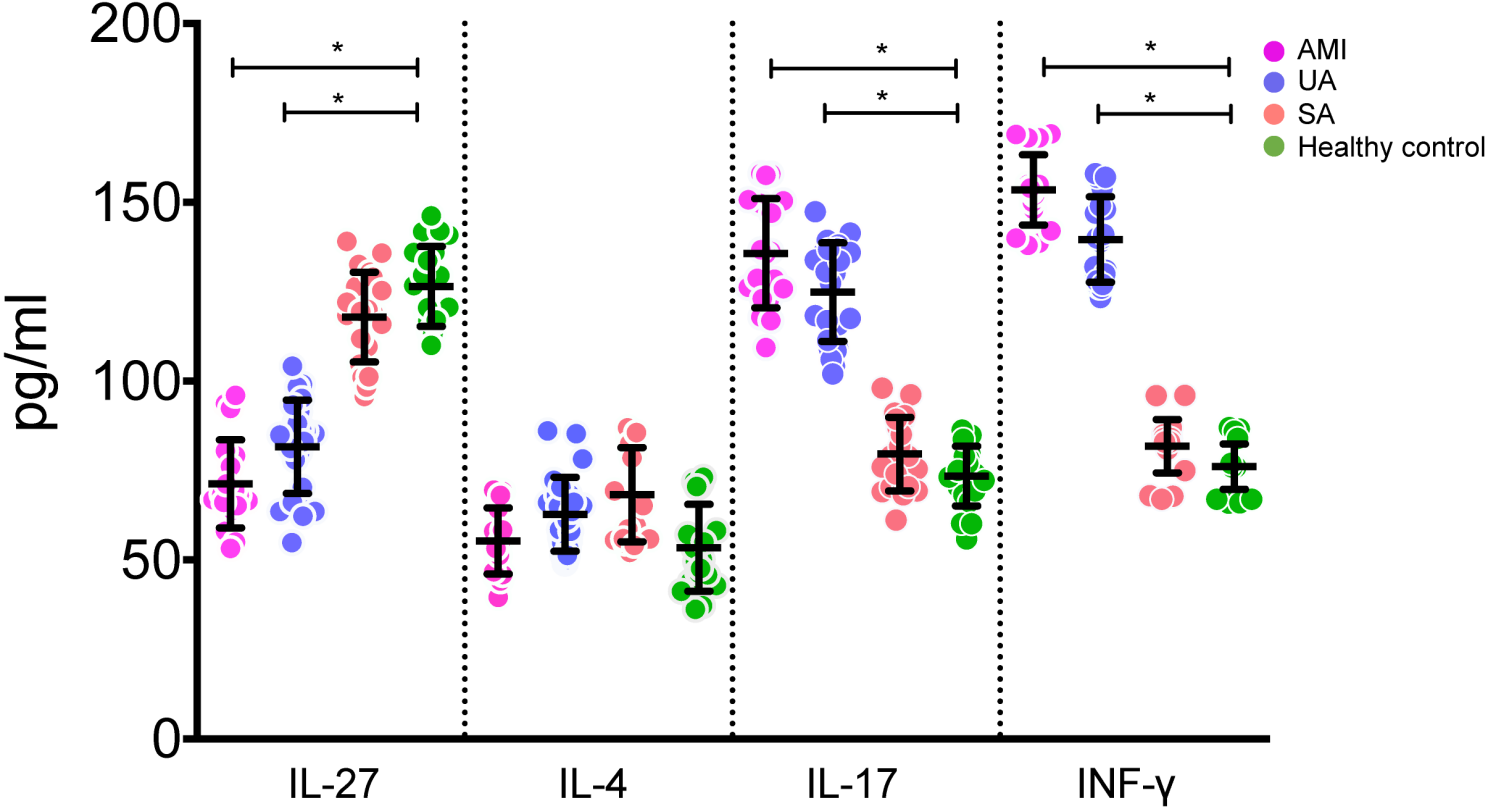
Levels of IL-27, IL-4, IL-17 and INF-*γ* in serums of patients in different groups.

Serum IL-27 levels in the AMI and UA groups were statistically significantly lower than those in the control and SA groups (all *P* < 0.05, Fig. 1). On the other hand, serum levels of IL-17 and IFN-γ in the AMI and UA groups were statistically significantly higher than those in the control and SA groups (*P* < 0.05, Fig. 1). There was no statistically significant difference in serum IL-4 level among different groups (*P* > 0.05, Fig. 1).

### Th2 cells and Th17 cell percentages in different ACS groups

**Figure 2:**
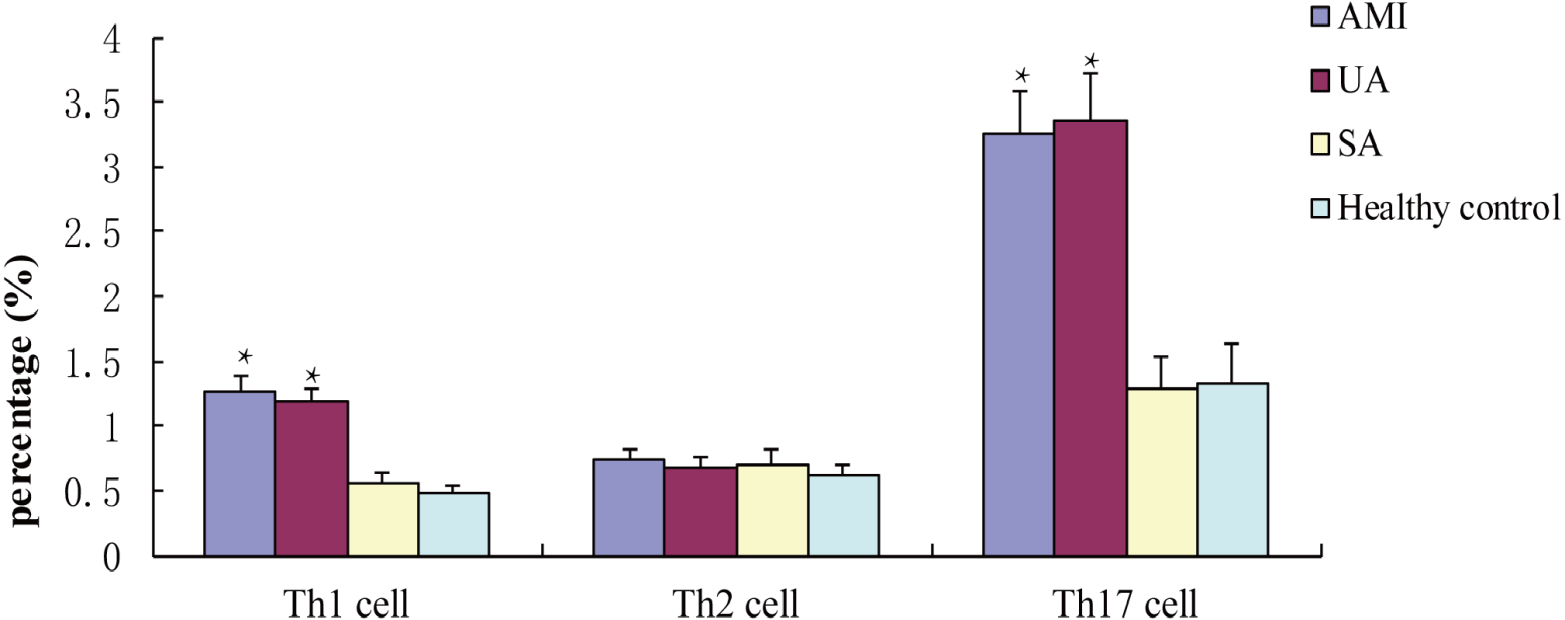
Percentages of TH1, Th2 and Th17 cells in different groups after incubated with PMA for 4 h.

Flow cytometry showed that percentages of Th1 cells and Th17 cells in patients in the AMI and UA groups were statistically significantly higher than those in the SA and control groups (*P* < 0.05, Fig. 2). The proportion of Th2 cells was not significantly different among the study groups (*P* > 0.05, Fig. 2).

### Influence of IL-27 stimulation on percentages of Th1 and Th17 cells

**Figure 3:**
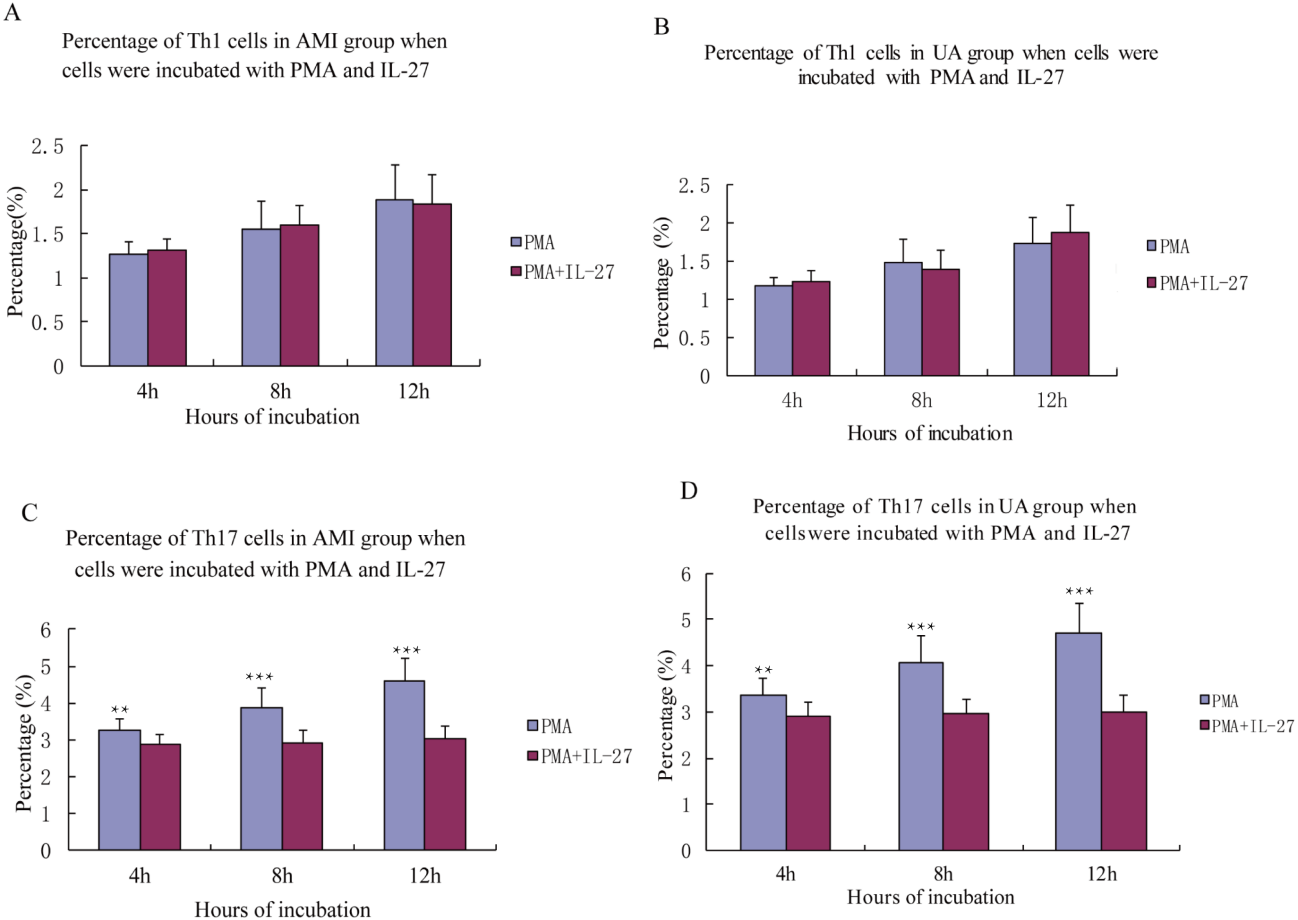
Percentage of Th1, Th17 cells in AMI, UA group when cells were incubated with PMA in IL-2.

IL-27 had no significant effect on levels of Th1 cells in the AMI and UA groups (all *P* > 0.05, Fig. 3). IL-27 stimulation induced Th17 cells in patients with AMI and UA (all *P* < 0.05, Fig. 3).

## Discussion

The incidence of cardiovascular disease is rising worldwide, and ACS is a major threat to human health. Recently, more attention has been paid to the role of inflammation in the progression of ACS. Inflammation participates in the development of coronary atherosclerosis, coronary stenosis, cardiomyocyte ischaemia, hypoxia, and necrosis, and myocardial infarction (Ren et al., 2017; Tang et al., 2017; Yin et al., 2014). IL-27 is an important inflammatory factor and plays a very important role in many diseases, including coronary heart disease (Al Shahi et al., 2015; Garg, Trout & Spector, 2017; Petes et al., 2017). Therefore, determining the levels of IL-27 and inflammatory cells in ACS patients could help reveal the role of IL-27 in the progression of ACS.

T lymphocytes comprise several subgroups, such as Th1, Th2, and Th17, which mediate T cell-dependent cellular and humoral immunity. Th1 cells secrete IFN-γ and tumour necrosis factor, which play important roles in cellular immunity *via* the induction of tumour cell apoptosis, inhibition of angiogenesis, and activation of antitumour activity (Gao et al., 2012). IL-4 is produced by Th2 cells and IL-17 is produced by Th17 cells. Both of them can regulate immune and inflammatory responses, and regulate the stability of the environment and the progression of autoimmune diseases in some tissues (Ribeiro et al., 2017).

Therefore, we evaluated the peripheral blood IL-27, IL-4, IL-17, and IFN-γ levels in patients with AMI, UA, SA, and healthy controls. Our results showed that the serum IL-27 levels in patients with AMI and UA were significantly lower than in the control and SA groups. Serum levels of IL-17 and IFN-γ in AMI and UA groups were higher than those in control and the SA groups. Our results suggested that the decrease in IL-27 may be an important factor in the development of ACS. Th1 and Th17 cells, which secrete large amounts of IFN-γ and IL-17, were increased in patients with AMI or UA. These results indicate that proliferation of Th1 cells and Th17 cells may promote the occurrence and development of ACS. Hashmi & Zeng (2006) found that IL-17 activity in UA and AMI was increased, indicating that IL-17-induced an inflammatory response in unstable ACS patients. This is consistent with the present results. Jafarzadeh, Nemati & Rezayati (2011) showed that serum IL-27 levels were significantly increased in patients with coronary heart disease compared to those in healthy controls, in contrast with our results. A recent study evaluated the serum level of IL-27 in ACS patients and concluded that IL-27 was independently related to impaired cardiac function and worse long-term prognosis, which raised the doubt whether IL-27 can be used as a biomarker of ACS (Grufman et al., 2018). In line with this, Miura et al. (2017) found plasma IL-27 levels were high in patients with CAD, which is not contradictory with us since they focused on CAD, but AMI and UA patients were evaluated in our study. However, such results again imply that IL-27 level is unlikely a biomarker for ACS, although further and larger patient cohorts are needed to confirm such conclusion. A previous study suggested that Th1 cells could promote the development of ACS (Lu et al., 2013). The exact role of IL-27 in patients with ACS remains unclear.

To verify our results, we further measured peripheral blood levels of IFN-γ, IL-4, and IL-17 in ACS patients using flow cytometry to reflect Th1, Th2, and Th17 cells. Results showed that the proportions of Th1 and Th17 cells in patients with AMI or UA were significantly higher than those in the SA and control groups, while the proportion of Th2 cells was not statistically significantly different among groups. This result is consistent with the results using ELISA, indicating that Th1 and Th17 cells are highly expressed in ACS patients and Th2 cells might not participate in the development of ACS. When we incubated cells with IL-27, we found that the ratios of Th17 cells in AMI and UA groups were statistically significantly reduced. The recent study suggested PMA stimulation of IL-27-treated M-Mac could enhance ROS production, and we speculated that PMA and IL-27 co-stimulation lowered the Th17 cell proportion through the same pathway (Garg, Trout & Spector, 2017).

## Conclusions

In conclusion, serum IL-27 level was lower, while Th1 and Th17 cells were higher in ACS patients. In addition, IL-27 could inhibit Th17 cell proliferation. The decrease in IL-27 levels could have been caused by the inhibition of Th17 cells, which further promoted the development of ACS. However, future studies are warranted to explore the mechanism of IL-27 in the regulation of T cells in patients with ACS.

##  Supplemental Information

10.7717/peerj.5652/supp-1 Data S1 Raw data

## Additional Information and Declarations

### Data Availability

The following information was supplied regarding data availability: The raw data are provided in Data S1.
